# Monthly Summary

**Published:** 1899-07

**Authors:** 


### Monthly Summary.

ANTISEPTIC USES OF OXYGENATED WATERS.—At the December meeting of the Paris Academy of Medicine several surgeons gave their experience in the use of oxygenated water in surgery. M. Lucas Championniere said that during the space of one year he had made clinical trial of this agent, and had found that oxygenated water was an antiseptic of remarkable power, and of special value in cases of suppuration of septic infection. Solutions of ten or twelve parts by volume seemed to act best and were very superior to solutions of perchloride of mercury. A solution of oxygen is not only capable of checking putrefaction, but it will also prevent its occurrence. In vaginal hysterectomy a preliminary douching of the canal with this solution gives better results than any other antiseptic fluid. In cases of septic infection after abortion, washing out the uterus with oxygenated water is just as satisfactory a method of treatment as curettage. In addition to the properties already mentioned, oxygenated water is hemostatic. On the other side, M. Charpentier argued that the liquid would not keep, and, moreover, was an admirable culture medium for strepococcus. M. Ferrand also spoke against it, saying the peroxide of hydrogen was not only an irritant but actually a caustic. M. Monod said that, in his opinion, nothing was better than carbolic spray, and M. Labbe agreed with him. M. Laborpe spoke in his turn in defense of the oxygen solution, saying that it was absolutely harmless and could be used as an intravenous injection. In reply, M. Championniere, while allowing that the solution in question was very unstable, argued that it could be obtained from the manufacturer in a state of great purity. and was certainly far superior to phenol.—*London Lancet*, December 17, 1898.

FORMALDEHYDE GAS.—Dr. H. E. Wood states that the chief interest in this drug centers in its powers as a germicide and disinfectant. As a gas it will penetrate not only animal tissues, but almost all organic substances, so that books infected with various pathogenetic germs could be disinfected by being shut up for fifteen minutes in an atmosphere containing the vapors of commerical formaline (40 per cent. aqueous solution of formaldehyde), one part to three hundred of air. The method of Trillat is the preferable one, and consists in the use of the formaldehyde directly after its production by the passage of the vapors of methylic alcohol over red hot metal. Kinyoun has shown that none of the ordinary fabrics are injured by the gas, which is capable of completely disinfecting curtains, carpets, clothing, bed covering, and the minor forms of furniture, although it is doubtful whether heavy upholstered furniture, such as sofas and mattresses, can, in their interior, be thoroughly disinfected. The gas is so irritating that no one can remain in the room during the disinfection, but the lamp employed is automatic, and can be left to itself. It can also be used for the removal of foul odors; one-half to one per cent. solution is sufficient for cleansing vessels in the anatomical laboratory. If the hands be washed with it and afterward with alcohol, they are rendered completely antiseptic, but are not stained or irritiated. It does not affect instruments, and it is efficient in preparing catgut and surgical dressing. For the cleaning of an enfeebled wound, a two per cent. solution is used; but for a continuous local application or free irrigation, one-fourth of one per cent. is sufficient.—*University Med. Magazine.*

---

EXTRACTING SUPERIOR CUSPIDS.—Sometimes the upper canines are very difficult to extract. When extracting the upper teeth for a plate, remove the teeth on each side of the canine and grasp it at the sides. The sides being more flat, the forceps will not slip, and the tooth can be easily rotated.—Otis Trotter, D. M. D., in "Pacific Medico-Dental Gazette."

ORTHOFORM.—Kallenberger ("Brit. Med. Jour.") finds that orthoform has the following properties :

1. It acts as a local anæsthetic wherever sensory nerve endings are exposed.

2. It is non poisonous, so that as much as 60 grn. was used in one week upon a large raw surface.

3. It is antiseptic.

Cases illustrating the value of orthoform are : (1) Fresh wounds, (2) burns, (3) ulcers of the legs, (4) carcinomatous ulcers, (5) syphilitic ulcers, and (6) toothache where there are exposed nerve endings. The pain mostly disappeared in from three to five minutes, after which the local anæsthesia was complete and lasted on an average for thirty-five hours. If the exudation is very abundant an ointment should be used instead of the powder, which may be washed away. In a case of ulcer of the leg where iodoform was substituted for the orthoform there was no return of the pain for seven hours. This period of freedom from pain is more marked the more prolonged the previous application of the orthoform has been. This agent also limits the exudation. Orthoform has been used internally in laryngeal ulcers, as well as in gastric ulcer and carcinoma. A chloride in addition to the base has been thus employed by Neumayer, but for surgical purposes the chloride is unsuitable, owing to its irritating properties.

---

NEVER attempt to crown or bridge on roots defective at apex; if you do, it will be safe to predict failure and evil results will follow in a limited period, and patients will realize that there was dishonesty or want of good judgment and skill, either of which will prove hurtful to the dentist and lowering to the profession.—Dr. B. F. Arrington, *Dental Weekly.*

---

CARBOLIC ACID POISONING.—Wash out the stomach with equal parts of vinegar and water; strychnine hypodermically.—A. Paget Steavenson, in "Dental Review:"

CREOSOTE AND SUNLIGHT.—If you hope to retain the full strength and medicinal properties of creosote, it must be kept in a bottle of white glass, sealed with a glass stopper, and placed where it may receive the light of the sun. If placed in dark bottle and hidden from daylight it deteriorates and loses its desired qualities.—Translated by B. J. Cigrand, for "Dental Digest."

---

TO REMOVE GUM TISSUE FROM A TOOTH CAVITY.—A difficulty which is frequently met with is the intrusion of the gum into the cervical edge of the cavity, so as to render difficult and very painful the adjustment of the rubber; the excess of gum may be burnt away with a minimum of pain by means of sodium of ethylate.—Robertson, "Dental Record."

---

BEAR THIS IN MIND.—If you are about to examine a septic case or one where you suspect syphilis, wash your hands in vinegar or dilute acetic acid, and you will soon discover by the smarting any little scratches or abrasions in your skin which might become the starting point of infection. —"Surgical and Dental News."

---

TANNOFORM CEMENT.—A mixture of tannoform and formaldehyde has been used with great success in more than 1,000 cases, for capping amputated pulps. It is strongly antiseptic, easily applicable, and becomes immediately hard. It is also of good service in pulpitis but it discolors the teeth.—"Dzierzawski, Ref. from D. M. J. Z."

---

COLD ABSCESSES.—The method of choice in the treatment of cold abscesses is the injection of iodoform in oil, ten per cent. If this fails, use oxygenated water.—Redard, in "Dental Review."

TO REPLACE A BROKEN TOOTH.—Where a tooth or block has been broken from a vulcanite plate, it can very often be reliably repaired by drilling a cavity in the rubber, just under the tooth pins, having sufficient undercuts for the retention of the material. Then by filling these undercuts with amalgam freshly mixed, and filling the rest of the cavity, covering the pins with soft solder, scraps of Weston's or Watt's metal preferably. This is accomplished by holding the tooth in place with plaster, or with the index finger protected with a pad of asbestos, while with any small instrument that will serve as a soldering iron, the solder is melted, using muriate of zinc as a flux. The work will be quite stable as soon as the amalgam has had time to harden. The advantages of the amalgam is that it forms a base upon which the metal used as a solder will flow, and averting its tendency to ball up and pull away from the cavity walls in the vulcanite. The solder will form a good union with the amalgam.—DR. ATKINSON, *American Dental Weekly.*

---

AMOUNT OF GOLD USED.—In answer to a query, I received the statement from Morgan, Hastings & Co., that annually there are used in the United States 30,000 ounces of gold for filling teeth, which is a very conservative estimate.—F. J. Fesler, *Dental Digest.*

---

IT PAYS BETTER to be a dentist than an oculist. A man has thirty-two teeth and only two eyes.—"Dental Review."

